# Brain Activity during Slow-Wave Sleep Points to Mechanism for Memory

**DOI:** 10.1371/journal.pbio.0020037

**Published:** 2004-01-20

How does your brain pass the time while you're sleeping? If you've ever wrestled the demons of insomnia, you know what sleepless nights can do to your mental agility. Sleep cycles in mammals are characterized by two distinct, successive sleep stages: slow wave and rapid eye movement (REM). Both stages of sleep have uniquely associated electrical activity in the brain, which neuroscientists can measure by placing elec-trodes on the brain during sleeping and waking states. What neuroscientists can't easily measure is the purpose of these two sequential sleep stages.

The notion that sleep helps to improve memory was introduced over 80 years ago. Since then, several studies have demonstrated that sleep deprivation following the acqui-sition of a new memory strongly impairs its consolidation. Insight into the mechanisms underlying this effect came from the observation that neuronal activity patterns detected during waking reappear during ensuing sleep, suggesting that newly acquired “memory traces” may be replayed in the brain to solidify neural connections and thus “consolidate” memory. These reverberating patterns of activity have been observed in both mammals and birds, pointing to a very general biological phenomenon.

Still, the relationship between brain reverberation and memory consolidation remains unclear for a number of reasons. First, studies to date have observed only subtle, short-lived reverberations lasting less than an hour and can't explain the memory-disrupting effects of sleep deprivation applied several hours and even days after initial memory encoding. And since brain reverberation in mammals has only been investigated in the hippocampus and cerebral cortex, it is unclear whether the phenomenon is specific to this neural circuit or is a more general property of the brain. Furthermore, reverberation studies have so far relied on neural activity measured in animals that were highly trained on specific laboratory tasks and therefore may simply not be representative of the acquisition of new memories. Finally, experience-dependent neural reverberation has been detected in both phases of sleep as well as waking, but no quantitative comparison of the different contributions of each state has been established.

In a study designed to address these concerns, Sidarta Ribeiro and his colleagues at Duke University in Durham, North Carolina, recorded over a hundred neurons continuously over the course of the normal sleep--wake cycle in rats, focusing on four major forebrain areas that are essential for rodent-specific behaviors. Halfway through the recording time, animals were transiently allowed to explore four strictly novel objects, each of them designed to provide different spatial and sensory cues. The researchers found that in all the forebrain areas examined the neuronal firing patterns recorded when the rats initially explored the new objects reverberated for up to 48 hours after these objects were removed. The reverberation of neuronal activity sampled when rats explored familiar environs was insignificant. Reverberation was most significant during slow-wave sleep (a state that accounts for nearly 40% of a rat's life), decreased during waking periods, and was highly variable during REM sleep.

In this study, Ribeiro et al. demonstrate that long-lasting neuronal reverberation following novel waking experiences can occur in several forebrain sites and is strongly enhanced during slow-wave sleep. Because neuronal reverberations are sustained for long periods, this may provide a mechanism to recall and amplify memories until they are effectively stored. On the basis of differences observed between REM and slow-wave sleep in this and previous studies, the authors propose that the two phases of sleep play separate and complementary roles in memory consolidation. Thus, the two stages of sleep give the brain a chance to process the novel events of the day in peace.

**Figure:**
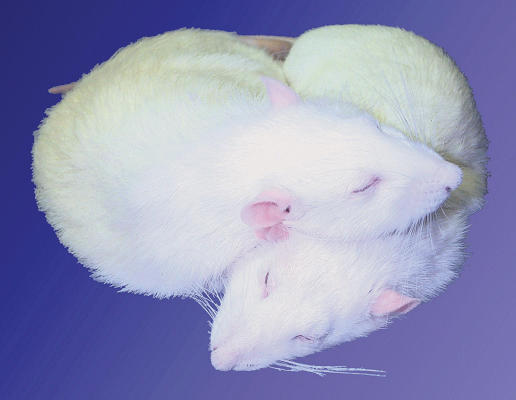
Sleeping rats

